# Isolation of large dense-core vesicles from bovine adrenal medulla for functional studies

**DOI:** 10.1038/s41598-020-64486-3

**Published:** 2020-05-05

**Authors:** Yelda Birinci, Julia Preobraschenski, Marcelo Ganzella, Reinhard Jahn, Yongsoo Park

**Affiliations:** 1 Department of Molecular Biology and Genetics, Koç University, Istanbul, 34450 Turkey; 2 Department of Neurobiology, Max-Planck-Institute for Biophysical Chemistry, Am Faßberg 11, 37077 Göttingen, Germany; 3 Neurological Disorders Research Center, Qatar Biomedical Research Institute (QBRI), Hamad Bin Khalifa University (HBKU), Qatar Foundation, PO Box 34110, Doha, Qatar

**Keywords:** Synaptic vesicle exocytosis, Molecular neuroscience, Neurotransmitters

## Abstract

Large dense-core vesicles (LDCVs) contain a variety of neurotransmitters, proteins, and hormones such as biogenic amines and peptides, together with microRNAs (miRNAs). Isolation of LDCVs is essential for functional studies including vesicle fusion, vesicle acidification, monoamine transport, and the miRNAs stored in LDCVs. Although several methods were reported for purifying LDCVs, the final fractions are significantly contaminated by other organelles, compromising biochemical characterization. Here we isolated LDCVs (chromaffin granules) with high yield and purity from bovine adrenal medulla. The fractionation protocol combines differential and continuous sucrose gradient centrifugation, allowing for reducing major contaminants such as mitochondria. Purified LDCVs show robust acidification by the endogenous V-ATPase and undergo SNARE-mediated fusion with artificial membranes. Interestingly, LDCVs contain specific miRNAs such as miR-375 and miR-375 is stabilized by protein complex against RNase A. This protocol can be useful in research on the biological functions of LDCVs.

## Introduction

Neurons communicate by neurotransmission, which involves vesicle fusion and neurotransmitter release^1,2^. Synaptic vesicles and large dense-core vesicles (LDCVs) participate in different functions in the nervous system^3,4^. Synaptic vesicles in the presynapse store classical neurotransmitters (e.g., glutamate, acetylcholine, GABA, and glycine), and mainly activate ion channels in the postsynaptic terminals, thereby transmitting electrical signals to the postsynapse. In contrast, LDCVs contain amines, neuropeptides, and other hormones that mainly stimulate G-protein-coupled receptors (GPCRs) to modulate synaptic responses^3–5^. LDCVs are minor components, amounting to only 1~2% when compared to synaptic vesicles in the central nervous system^6–8^, but they are highly abundant in specialized brain regions (such as the hypothalamus), in dopaminergic neurons and in sympathetic neurons of the peripheral nervous system^6^.

Chromaffin cells are modified sympathetic ganglia of the sympathetic nervous system^7,8^. Chromaffin cells are enriched with LDCVs, also called chromaffin granules, and therefore constitute a widely-used model system for in-depth study of LDCVs^9^. LDCVs of chromaffin cells contain catecholamines (i.e., adrenaline, noradrenaline, and dopamine), peptides (e.g., encephalin and neuropeptide Y), and proteins (chromogranins, secretogranins)^10^. Recently, we reported that LDCVs store microRNAs (miRNAs), which are released by vesicle fusion in response to stimulation, and miR-375 is the most abundant miRNA in LDCVs^11,12^.

A protocol to isolate LDCV from bovine adrenal medulla was reported in the 1950s^13^, then modified^14^ to include step density gradient centrifugation in the late 1960s. This protocol was widely used in many studies and formed the basis for establishing the proteomics of LDCVs^15^, and for identifying their content including amines, peptides, and proteins released by LDCV exocytosis^10^. However, the purity of LDCVs isolated by this protocol is limited, compromising biochemical and biophysical studies of isolated LDCV.

Here, we present a modified protocol for obtaining LDCVs with high yield and high purity from bovine adrenal medulla. The conventional methods for LDCV isolation use a sucrose step gradient of high density^16,17^. Upon centrifugation LDCVs migrate through these high density sucrose solutions (e.g., 1.6 M) whereas other organelles exhibiting lower buoyant density largely remain on top of the sucrose layer. However, the buoyant density of mitochondria overlaps slightly with that of LDCVs, so contamination with mitochondria is a serious problem. The protocol for LDCV isolation described here improves the purity by **i)** increasing the maximal sucrose concentration to 2.0 M and **ii)** applying a continuous sucrose density gradient.

**Figure 1:**
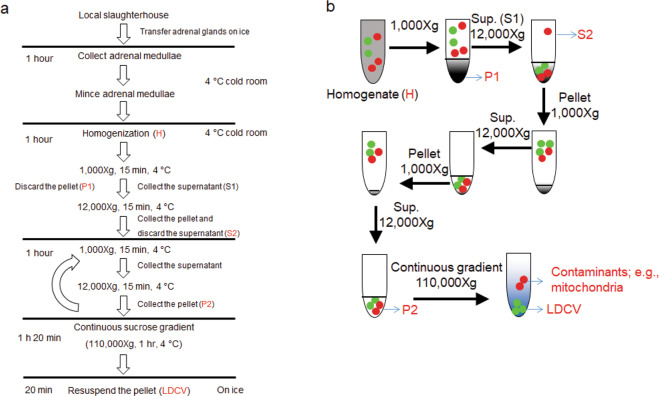
Schematic overview over the procedure for isolating LDCVs from bovine adrenal medulla. (**a**) Overview of timing and purification protocol to isolate LDCVs. (**b**) Schematic of LDCV isolation. Green: LDCVs; Red: contaminant organelles (e.g., mitochondria). Black, gray: cell debris.

**Figure 2:**
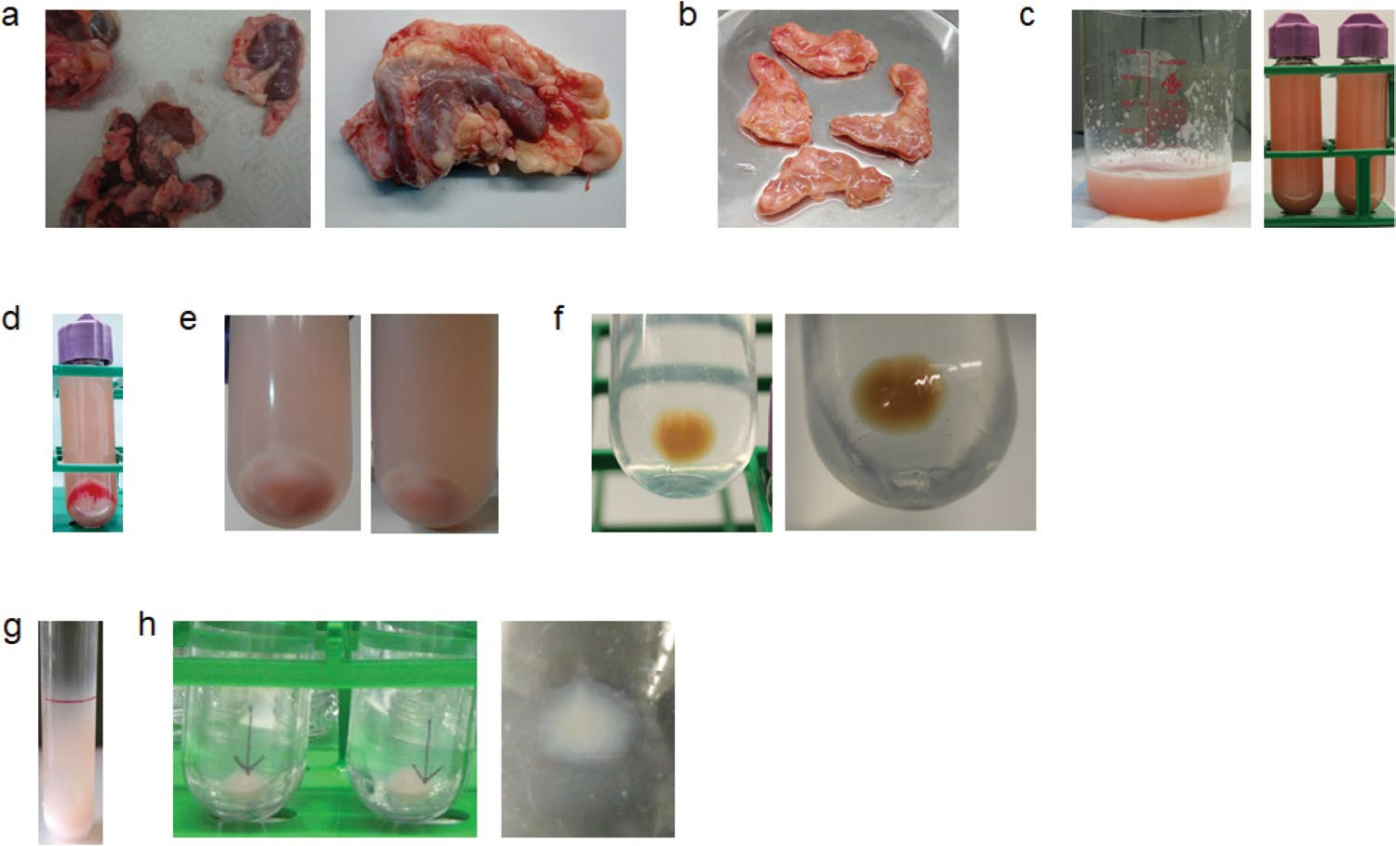
Photos of individual steps during the purification of LDCVs. (**a**) Adrenal glands transported on ice from local slaughterhouse. Note that adrenal glands are surrounded by fat. (**b**) Adrenal medullae after removal of adrenal cortex. **(c)** Homogenate (H) of adrenal medullae after homogenization. **(d)** Supernatant (S1) and pellet (P1) after centrifugation. **(e)** P2 fraction before and, **(f)** after washing. **(g)** LDCV distribution after continuous sucrose gradient. LDCVs are pink, which results from the presence of cytochrome b561 in the LDCV membrane^27^, whereas mitochondria are brown. Note that the pellet is pink and the supernatant is brown. **(h)** After removing the supernatant, pellet are LDCVs.

**Figure 3:**
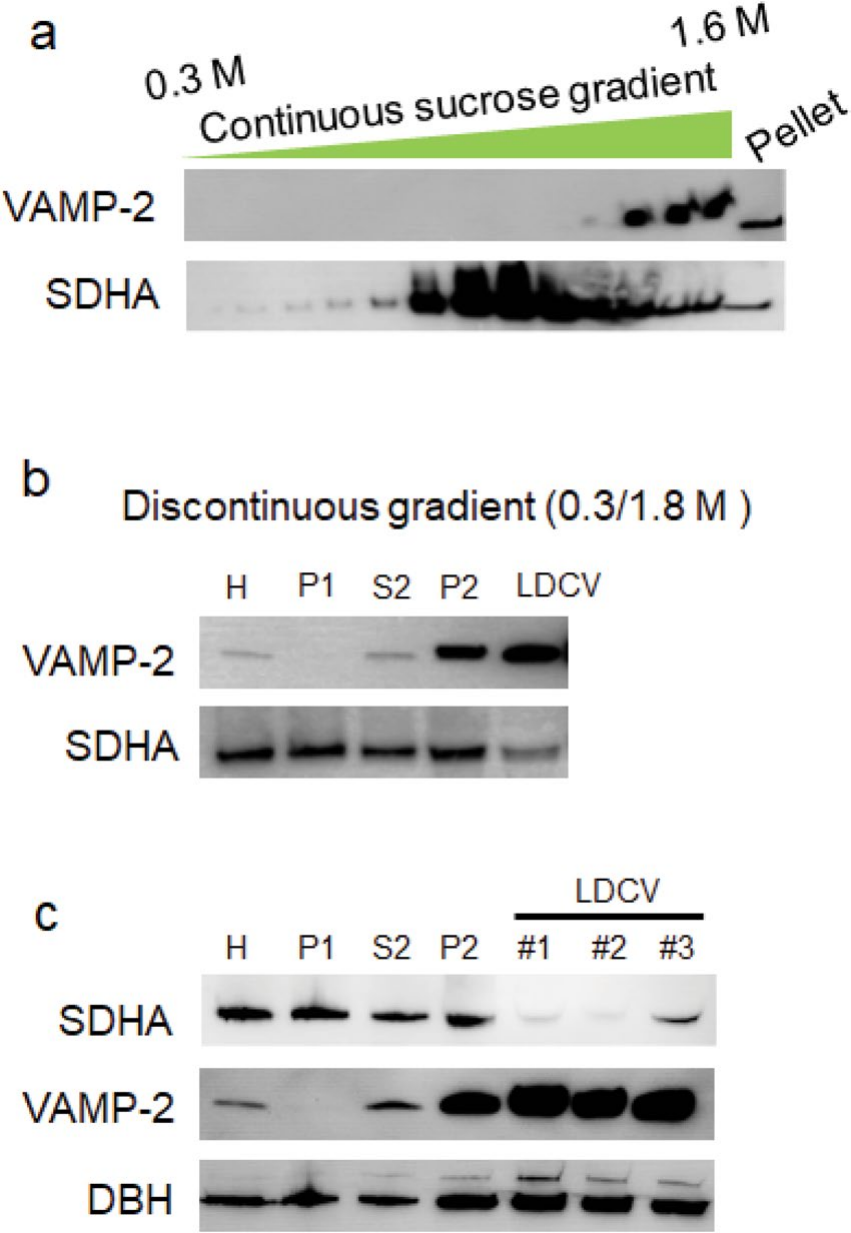
Separation of LDCVs from mitochondria by increasing the maximal density. (**a**) Continuous sucrose gradient from 0.3 M to 1.6 M was used, which resembles conventional protocols. Profile of VAMP-2 (a marker for LDCV membranes) and succinate dehydrogenase complex subunit A (SDHA, a marker for mitochondria) across gradient fractions. Aliquots of equal volume from each fraction were subjected to SDS-PAGE and immunoblotting, as described elsewhere^18^. Mitochondria (SDHA) contaminants were present throughout the gradient and are still significant in the pellet fraction containing the LDCVs. (**b**) The LDCV fraction was recovered from the pellet after a discontinuous sucrose gradient in which a 1.8 M sucrose cushion was overlaid with 0.3 M sucrose. 20 μg of proteins from each fraction were subjected to immunoblotting. Homogenate (H), the pellet containing nuclei and cell debris (P1), second supernatant (S2), crude LDCV (P2), large dense-core vesicle (LDCV). (**c**) Mitochondrial contamination of LDCV pellets varies with the type of sucrose gradient: **#1**: continuous sucrose gradient (0.3 M/1.9 M), **#2**: continuous sucrose gradient (0.3 M/2.0 M), **#3**: discontinuous gradient (0.3 M/2.0 M). SDHA (marker for mitochondria). VAMP-2 and dopamine-beta-hydroxylase (DBH) are markers for LDCVs. Full-length blots are presented in the Supplementary Information.

**Figure 4:**
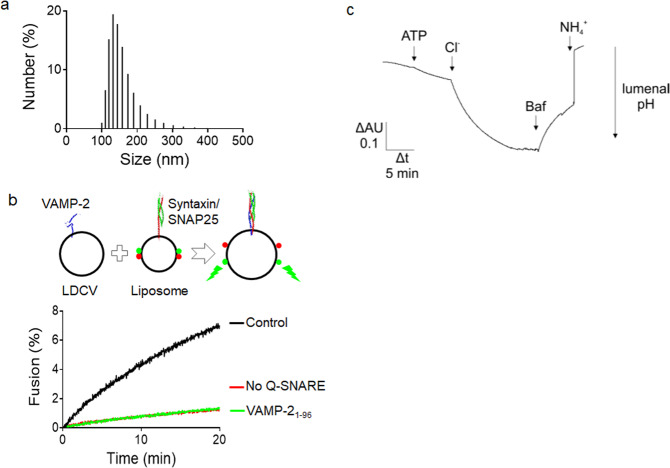
Biophysical and biochemical properties of purified LDCVs. (**a**) Size distribution of purified LDCVs analyzed using dynamic light scattering. The histogram shows the numbers as percentage of total. Average diameter of LDCVs is 153.7 nm, with a standard deviation (SD) of 42.2 nm. (**b**) Fusion of LDCVs with liposomes containing SNARE acceptor complexes. Fusion was measured by dequenching of labeled membrane lipids (lipid mixing)^18,22,28^ (top). Plasma membrane-mimicking liposomes contain phospholipids labelled with NBD (green fluorescence) and rhodamine (red fluorescence). The stabilized Q-SNARE complex (syntaxin-1A and SNAP-25A) called the deltaN complex^23^ is reconstituted in liposomes that mimic the plasma membrane. Fluorescence resonance energy transfer (FRET) between the two fluorophore-labeled lipids is reduced after LDCV fusion due to lipid dilution by unlabeled lipids of LDCVs, thus de-quenching the donor fluorescence. Soluble synaptobrevin-2 (VAMP-2_1-96_) and omission of SNAREs in liposomes prevented lipid mixing (bottom). Fluorescence values are normalized as a percentage value of the maximum donor fluorescence induced by 0.1% (vol/vol) Triton X-100 (TX-100) detergent treatment at the end of experiments. (**c**) ATP and Cl^-^ dependent acidification in LDCVs. LDCV acidification was monitored using 1 mM acridine orange as a reporter dye. The reaction was started by the addition of 1.2 mM MgATP followed by 50 mM KCl. Addition of 0.2 µM of the V-ATPase inhibitor Bafilomycin results in luminal re-alkalinization, indicating V-ATPase-specific proton pumping. The reaction was stopped by adding 15 mM (NH_4_)_2_SO_4_ which equilibrates the luminal pH with that of the medium.

**Figure 5:**
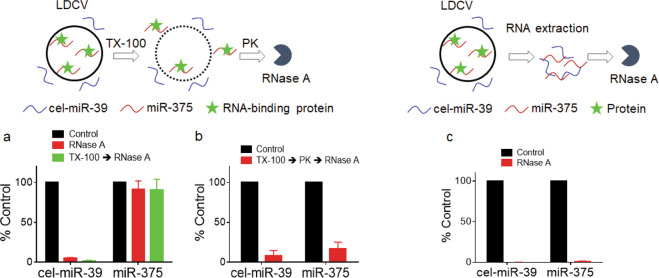
Detection of miR-375 stored in LDCVs. (**a**) Schematic of test for miR-375 stored in LDCVs (top); 1% (vol/vol) TX-100 was incubated with LDCVs to disrupt the vesicle membrane. RNase A was then applied to degrade miR-375. Relative levels of miR-375 with or without 1% (vol/vol) TX-100 were determined by qRT-PCR (bottom). (**b**) LDCVs were treated with Proteinase K in the presence of TX-100, followed by RNase A treatment. (**c**) RNase A was applied after RNA extraction using the miRNeasy Mini Kit. Proteins are removed from RNA samples. Values represent a percentage of control and data are mean ± SD from three to four independent replicates.

The protocol outlined and illustrated (Figs. 1 and 2) for LDCV isolation yields highly pure LDCVs, mostly excluding mitochondria (Fig. 3). Purified LDCVs retain activity in V-ATPase-dependent acidification as well as in SNARE-mediated membrane fusion (Fig. 4). miR-375, the most abundant miRNA stored in LDCVs, remains stable even in the presence of RNase A. We provide evidence showing that miR-375 can be stabilized by protein complex against the degradation by RNase A (Fig. 5). This protocol will be useful in research on the biological functions of LDCVs, including vesicle fusion, vesicle acidification, monoamine transport, and the functions of miRNAs stored in LDCVs.

## Results

### LDCV isolation

LDCVs were isolated from bovine adrenal medulla by combining differential and continuous sucrose gradient centrifugation; schematic overview and photos of individual steps during the purification are illustrated in Figs. 1 and 2. The quality of purified LDCVs can be checked using immunoblotting^18^ (Fig. 3). We first tested the purity of LDCVs isolated using the conventional protocols. The conventional methods applied a sucrose step gradient of high density, which exploits the fact that LDCVs have the highest known buoyant density of all organelles^16,17^. Smith and Winkler used 0.3 M/1.6 M sucrose gradient to purify LDCVs^14^ and we observed that the continuous sucrose gradient from 0.3 M to 1.6 M gives rise to the serious contamination of mitochondria (Fig. 3a). VAMP-2 and succinate dehydrogenase complex subunit A (SDHA) as protein markers represent the presence of LDCV and mitochondria, respectively. LDCVs migrate into the pellet fraction due to their high buoyant density, whereas other organelles largely remain on top of the sucrose layer. We only collected the pellet fraction for LDCVs after continuous sucrose gradient centrifugation. However, the buoyant density of mitochondria overlaps slightly with that of LDCVs. Mitochondria contaminants (SDHA) are present throughout the gradient and are still significant in the pellet fraction containing LDCVs in the conventional method that use 0.3 M/1.6 M sucrose gradient (Fig. 3a).

We next tried discontinuous sucrose gradient centrifugation with 0.3 M/1.8 M sucrose to isolate LDCVs (Fig. 3b). Mitochondria (SDHA) is still present in the pellet together with LDCVs; LDCVs are from the pellet fraction, proposing that 0.3 M/1.8 M sucrose gradient method is limited for isolating LDCVs with high purity. Then we examined mitochondrial contamination from LDCV pellets using the different sucrose gradient (Fig. 3c): i.e., **#1:** continuous sucrose gradient (0.3 M/1.9 M), **#2:** continuous sucrose gradient (0.3 M/2.0 M), **#3:** discontinuous gradient (0.3 M/2.0 M). SDHA is mostly removed, whereas VAMP-2 and dopamine-beta-hydroxylase (DBH), markers for LDCVs, are highly enriched in the continuous sucrose gradient with 0.3 M/2.0 M compared to the discontinuous sucrose gradient (Fig. 3c), suggesting that continuous sucrose gradient is much better for the high purity than discontinuous sucrose gradient. Taken together, we improved the purity of LDCV by **i)** increasing the maximal sucrose concentration to 2.0 M and **ii)** applying a continuous sucrose density gradient. This protocol is optimized to improve the purity of LDCVs by removing mitochondrial contamination.

### Dynamic light scattering analysis

Dynamic light scattering analysis was carried out to determine the size distribution of isolated LDCVs from bovine adrenal medulla, which give rise to 153.7 ± 42.2 nm (Fig. 4a). The diameter of purified LDCVs can also be measured using electron microscopy that showed a heterogeneous size distribution with an average diameter of 167.7 nm^18^, correlating with dynamic light scattering analysis (Fig. 4a). The average diameter of purified LDCVs with ~153 nm is smaller than that of total LDCVs in chromaffin cell; the average diameter is 356 nm^19^. It is because this method with 0.3 M/2.0 M continuous sucrose gradient purifies mainly mature LDCVs (smaller in size) whereas immature LDCVs are separated away^18^. Mature LDCVs become smaller and condensed through removal of water during the maturation process^20,21^, proposing that isolated LDCVs have high purity and mature LDCVs are mainly purified^18^.

### Vesicle fusion assay using purified LDCVs

LDCVs frozen for several months are structurally and functionally intact as judged by functional assay. We measured the activity with respect to SNARE-mediated membrane fusion. The reconstitution of LDCV fusion was previously established using purified LDCVs^18,22^. Purified LDCVs fused with plasma membrane-mimicking liposomes that contain the stabilized soluble *N*-ethylmaleimide-sensitive factor attachment protein receptor (Q-SNARE) complex with high efficiency (Fig. 4b). Plasma membrane-mimicking liposomes containing NBD- and rhodamine-labeled phospholipids were reconstituted with the stabilized acceptor complex known as the ΔN complex: a preformed complex of syntaxin-1A (lacking the N-terminal Habc domain) and SNAP-25A containing the C-terminal fragment of VAMP-2 (residues 49–96)^23^. Liposomes contained 45% phosphatidylcholine, PC; 15% phosphatidylethanolamine, PE; 10% phosphatidylserine, PS; 25% cholesterol; 4% phosphatidylinositol, PI and 1% phosphatidylinositol 4,5-bisphosphate, PI(4,5)P_2_. Fusion was monitored by a lipid-mixing assay in which fluorescence resonance energy transfer (FRET) between the two fluorophore-labeled lipids become reduced due to lipid dilution after vesicle fusion, thus causing de-quenching of the donor fluorophore^24^. LDCV fusion is SNARE-dependent^18,22^, because soluble VAMP-2 or lack of SNARE proteins in plasma membrane-mimicking liposomes completely block LDCV fusion (Fig. 4b).

### Vesicle acidification by V-ATPase

Another functional assay for intact LDCVs is to test the activity with respect to luminal acidification. A key role of LDCVs is the accumulation of monoamines such as adrenaline, noradrenaline, dopamine and serotonin prior to their release through SNARE dependent exocytosis. Monoamine loading into LDCVs relies on a proton electrochemical gradient (ΔμH^+^) generated by the V-ATPase in the LDCV membrane. In presence of ATP the V-ATPase pumps protons into the vesicle lumen which results in the formation of a proton gradient (ΔpH) and an inside positive membrane potential (ΔΨ). In absence of negative counter ions ΔpH is comparably small, as the V-ATPase is slowed down by the accumulation of positive charges in the vesicle lumen. However, in presence of membrane permeable anions such as Cl^−^, anion co-influx neutralizes the positive charges, which stimulates proton pumping and thus facilitates lumenal acidification. Indeed, we observed an initial ATP dependent acidification that was strongly enhanced upon addition of Cl^−^ (Fig. 4c), demonstrating that the purified LDCVs exhibit a pronounced proton pumping activity. Furthermore, blocking of the V-ATPase with the inhibitor Bafilomycin reversed acidification, suggesting V-ATPase-specific acidification.

### Stability of miR-375 stored in LDCVs

We have previously reported that LDCVs contain a variety of non-coding RNAs including miR-375, which is a dominant miRNA in LDCVs^11^. miR-375 is protected in the presence of RNase A as reported previously^11^. Here we tested how miR-375 can be stabilized after exocytosis. The presence of miRNAs in LDCVs are shown using qRT-PCR (Fig. 5) and the details of experiment set-up is described in Table 1. A synthesized *Caenorhabditis elegans* miRNA (cel-miR-39) as the spike-in control was added to normalize the RNA extraction efficiency. RNase A is not able to pass across the membranes so that it selectively degrades vesicle-free miRNA, but not vesicle-incorporated miRNA. RNase A degraded cel-miR-39, the spike-in control, but miR-375 remained intact, suggesting that miR-375 is incorporated in LDCVs^11^.

**Table 1:** Tests for the stability of miR-375 stored in LDCVs.

Treatment Reactions	Control		+RNase A		+TX-100 + RNase A		+TX-100 + Proteinase K + RNase A	
	Vol.	Final Conc.	Vol.	Final Conc.	Vol.	Final Conc.	Vol.	Final Conc.
LDCVs	5 µl	50 µg	5 µl	50 µg	5 µl	50 µg	5 µl	50 µg
Syn-cel-miR-39	5 µl	0.5 pmol	5 µl	0.5 pmol	5 µl	0.5 pmol	5 µl	0.5 pmol
RNase-DNase Free PBS	10 µl		7.5 µl		5.5 µl		6 µl	
10% TX-100 (vol/vol)					2 µl	1%	2 µl	1%
Proteinase K(1 mg/ml)							2 µl	100 µg/ml
Protease inhibitor (2×)							10 µl	
RNase-DNase Free PBS							5 µl	
RNase A (100 µg/ml)			2 µl	10 µg/ml	2 µl	10 µg/ml	4 µl	10 µg/ml
RNase inhibitor (40 U/µl)			0.5 µl	1 U/µl	0.5 µl	1 U/µl	1 µl	1 U/µl
Final Volume	20 µl		20 µl		20 µl		40 µl	

Next, we tested if miR-375 can be degraded by RNase A after Triton X-100 (TX-100) detergent treatment. TX-100 is a non-ionic detergent and completely disrupts the vesicle membrane. cel-miR-39 was mixed with LDCVs prior to TX-100 and then RNase A was applied after TX-100 treatment (Fig. 5a). We observed that miR-375 remains resistant to RNase A even in the presence of TX-100, whereas cel-miR-39 becomes degraded by RNase A (Fig. 5a). Then, we hypothesized that proteins might stabilize miR-375 and miR-375 was degraded by RNase A when proteinase K (PK) was included in TX-100-treated LDCVs (Fig. 5b). To confirm that proteins can protect miR-375, RNase A is applied after RNA extraction from protein complex using miRNeasy Mini Kit. Indeed, miR-375 is completely degraded by RNase A after proteins are removed; this result suggests that miR-375 is stabilized by proteins (Fig. 5c). Altogether, this protocol provides methods to test the presence of miRNAs in LDCVs and the stability of miR-375.

## Discussion

This protocol obtains intact LDCVs from bovine adrenal medulla. These purified LDCVs can then be used to study their composition and content and to investigate their biochemical features such as proton pumping and SNARE-mediated membrane fusion as described in Fig. 4. More interestingly, purified LDCVs may be carriers in neurons and neuroendocrine cells that deliver miRNAs and other non-coding RNAs to extracellular fluid. miR-375 is stored in LDCVs and stabilized by protein complex (Fig. 5).

The protocol of LDCV isolation begins with mild homogenization of bovine adrenal medulla to disrupt the plasma membrane and release free LDCVs into the supernatant. These extracts are then loaded on a continuous sucrose density gradient ranging from 0.3 M to 2.0 M and centrifuged, resulting in the separation from other organelles. Mitochondria (marker, SDHA), late endosomes/multivesicular bodies (LEs/MVBs) and lysosomes (marker, LAMP-1), early endosomes (marker, EEA-1), endoplasmic reticulum (marker, calnexin), proteasomes (marker, Rpt-4), and peroxisomes (marker, catalase) are mostly removed, whereas LDCVs are highly enriched^18^. This fractionation method yields primarily mature LDCVs with 100~200 nm in diameter^18^ whereas immature LDCVs (identified here by the presence of VAMP-4) are also removed. Due to the vesicle maturation and condensation, mature LDCVs exhibit much higher buoyant density than immature LDCVs, and thus mature LDCVs are mainly enriched in the pellet^18^, which is used for functional studies. Mitochondria contaminations in different conditions of sucrose density gradient are presented in Fig. 3c; e.g., continuous sucrose gradient ranging from 0.3 M/1.8 M to 0.3 M/2.0 M and discontinuous sucrose gradient (0.3 M/2.0 M). Continuous sucrose gradient with 0.3 M/2.1 M was also tried to isolate LDCVs, but LDCVs were barely isolated with very little pellet (data not shown); LDCVs are not able to migrate through high density sucrose solutions into the pellet due to high sucrose concentration in case of 0.3 M/2.1 M sucrose gradient, thereby leading to extremely low yield of LDCVs. We tested different sucrose gradient ranging from 0.3 M/1.6 M to 0.3 M/2.1 M and mitochondria are mostly removed in our protocol, when we use continuous sucrose gradient (0.3 M/2.0 M) (Fig. 3c). Furthermore, our previous overlay assay shows ~95% purity of isolated LDCVs^11^, supporting that most mitochondria are removed and isolated LDCVs are highly pure.

LDCVs contain a variety of miRNAs, which are released by LDCV exocytosis^11^. A new paradigm suggests that secreted miRNAs constitute novel neuromodulators by regulating cell−to−cell communication that include gene silencing and cellular signaling. We propose ‘ribomone (ribonucleotide + hormone)’, because miRNAs are stored inside the vesicles and released by active vesicle fusion in response to stimulation so that secreted miRNAs might regulate cell−to−cell communication including gene silencing and cellular signaling. Our data provide evidence that secreted miR-375 can be stabilized by protein complex against RNase A, implying that secreted miRNAs might function a physiological role for long-term with high stability.

In conclusion, this protocol improves the purity of LDCVs by combining differential and continuous sucrose gradient centrifugation. This technique can be useful in research on the biological functions of LDCVs, including vesicle fusion, vesicle acidification, monoamine transport, and the functions of miRNAs stored in LDCVs. The purification of LDCVs also contributes to study psychiatric and mental disorders, which might be caused by imbalance of serotonin, adrenaline, and dopamine, because LDCVs store most of monoamine transmitters such as serotonin, adrenaline, and dopamine, which are directly linked to psychiatric and mental disorders.

## Materials and Methods

### Materials

All reagents are available from Sigma-Aldrich unless otherwise stated and should be of analytical grade or higher. RNase A and RNase inhibitor, RiboLock were from Thermo Scientific. Protease inhibitor, cOmplete ULTRA Tablets, Mini, EDTA-free EASYpack was from Roche. Antibodies against VAMP-2 and DBH were from Synaptic Systems (Göttingen, Germany). Antibodies against SDHA was purchased from Abcam (Cambridge, MA). Lipids were from Avanti (Alabaster, AL). RNA isolation kit, miRNeasy Mini Kit (Qiagen, cat. no. 217004). cDNA synthesis kit, miScript RT II kit (Qiagen, cat. no. 218161). Synthetic *Caenorhabditis elegans* miR-39 (syn-cel-miR-39), spike-in control (Qiagen, cat. no. 219610). Synthetic miR-375 (Syn-bta-miR-375), miScript miRNA Mimic (Qiagen, cat. no. 219600). Primer for cel-miR-39, UCACCGGGUGUAAAUCAGCUUG (Qiagen cat. no. MS00019789). Primer for bta-miR-375; UUUUGUUCGUUCGGCUCGCGUGA (Qiagen cat. no. MS00053865). miScript SYBR Green PCR Kit (Qiagen, cat. no. 218073).

### LDCV isolation

Fresh bovine adrenal glands were obtained from a local slaughterhouse. After trimming away the cortex and fat, the medullae were minced with a scissor in 300 mM sucrose buffer (300 mM sucrose, 20 mM HEPES, pH 7.4 adjusted with KOH) and then homogenized using a cooled a Glass-Teflon homogenizer at 1,000 rpm (H, homogenate). PMSF (200 μM) was added to prevent protein degradation. All subsequent steps were carried out at 0°–4 °C. After centrifugation at 1,000 g for 15 min at 4 °C, the pellet containing nuclei and cell debris (P1) was discarded. The supernatant (S1) was further centrifuged (12,000 g, 15 min, 4 °C), followed by the additional cycle of resuspension and centrifugation for washing step. The resulting pellet (P2, crude LDCV fraction) was resuspended in 300 mM sucrose buffer and loaded on top of a continuous sucrose gradient (from 300 mM to 2.0 M) to remove other contaminants including mitochondria. LDCVs were collected from the pellet after centrifugation at 110,000 g for 60 min in a Beckman SW 41 Ti rotor and resuspended with the buffer (120 mM K-glutamate, 20 mM K-acetate, 20 mM HEPES.KOH, pH 7.4). The fraction directly on top of the pellet was removed and the pellet was only resuspended in order to purify mature LDCVs. The purified LDCVs can be snap-frozen in liquid nitrogen and stored for several months at −80 °C. Make small aliquots of LDCV samples to reduce damage by freeze-thaw cycles. Size distribution of purified LDCVs was determined using dynamic light scattering (NanoPlus DLS, Particulate Systems).

### Vesicle fusion assay

Vesicle fusion reactions were performed at 37 °C. 50 μg of purified LDCVs and 10 μl of plasma membrane-mimicking liposomes were mixed in 1 ml of buffer containing 120 mM K-glutamate, 20 mM K-acetate, 20 mM HEPES-KOH (pH 7.4). Plasma membrane-mimicking liposomes contained the stabilized Q-SNARE complex^23^. Fluorescence dequenching signal was measured with wavelengths of 460 nm for excitation and 538 nm for emission. Fluorescence values were normalized as the percentage value of the maximum donor fluorescence induced by 0.1% Triton X-100 detergent treatment at the end of experiments. Control represents basal fusion without any treatment.

### Immunoblotting

Glycerol-containing gels with 0.1% SDS were used to separate low molecular weight proteins with high resolution. Proteins were transferred to a nitrocellulose membrane and then blocked with 5% non-fat dry milk in solution (20 mM Tris-HCl, pH 7.4, 137 mM NaCl, and 0.1% tween-20). Proteins as indicated in manuscripts were detected using horseradish peroxidase-conjugated secondary antibodies.

### Vesicle acidification assay

Acidification measurements were performed as described previously using acridine orange (AO, Molecular Probes) as a pH sensitive dye^25^. Changes in absorbance at 492 nm (ΔAU) were monitored in an Aminco dual-wavelength spectrophotometer using absorbance at 530 nm as reference, giving a read-out of lumenal pH changes^26^. Usually, 600–650 µl buffer (300 mM glycine, 10 mM MOPS, pH 7.3, and 2 mM MgSO4) were mixed in a 1 ml glass cuvette with purified LDCVs containing 10 µM AO and measured at 32 °C. The measurements were performed in 300 mM glycine, 10 mM MOPS, 2 mM MgSO4, pH 7.3 buffer.

### Quantification of miR-375 using qRT-PCR

Several reactions to test the stability of miR-375 in the presence of RNase A, TX-100, and/or proteinase K was described in details in Table 1. 0.5 pmol of syn-cel-miR-39 was incubate with ~50 µg of LDCVs for 5 min at RT (~22 °C). 1% (vol/vol) Triton^Tm^ X-100 (TX-100) was treated with LDCVs for 5 min at room temperature. Then, 10 µg/ml (final concentration) of RNase A was applied for 15 min in the presence or absence of TX-100. If RNase A is used, then 1 U/µl RNase inhibitor was added for 5 min to stop the RNase activity. In cases of proteinase K treatment 2 µl of 100 µg/ml of proteinase K was applied for 30 min. A protease inhibitor cocktail was added for 5 min to stop proteinase activity. Then 10 µg/ml RNase A was applied to degrade RNA.

Total RNA from LDCV samples with different treatments (Table 1) was isolated using miRNeasy Mini Kit according to the manufacturer’s protocol. Isolated RNAs were transferred into 0.2 ml PCR Strip tubes, then cDNA syntheses was performed using miScript RT II kit according to the manufacturer’s protocol. 5X miScript HiSpec buffer was used to prepare the reaction mix. miR-375 and cel-miR-39 were quantified using qRT-PCR according to miScript SYBR Green PCR Kit protocol. For miRNA quantification of miR-375 and cel-miR-39, a standard curve should be established using syn-bta-miR-375 and syn-cel-miR-39. For this task, serial dilutions (0.02 pM to 2 nM) of syn-bta-miR-375 and syn-cel-miR-39 were prepared. qRT-PCR analysis was performed using Bio-Rad, CFX Connect™ Real-Time PCR Detection System. Crossing point-PCR-cycle (Cp) values were used to plot the standard curve and to analyze the concentraction of miR-375 stored in LDCVs and cel-miR-39, which is a spike-in control.

## Supplementary information

Supplementary information.

## Data Availability

The datasets generated during the current study are available from the corresponding author on reasonable requests.
