# Author Correction: Isolation of large dense-core vesicles from bovine adrenal medulla for functional studies

**DOI:** 10.1038/s41598-022-21361-7

**Published:** 2022-10-06

**Authors:** Yelda Birinci, Julia Preobraschenski, Marcelo Ganzella, Reinhard Jahn, Yongsoo Park

**Affiliations:** 1 Department of Molecular Biology and Genetics, Koç University, Istanbul, 34450 Turkey; 2 Department of Neurobiology, Max-Planck-Institute for Biophysical Chemistry, Am Faßberg 11, 37077 Göttingen, Germany; 3 Neurological Disorders Research Center, Qatar Biomedical Research Institute (QBRI), Hamad Bin Khalifa University (HBKU), Qatar Foundation, PO Box 34110, Doha, Qatar

Correction to: *Scientific Reports* https://doi.org/10.1038/s41598-020-64486-3, published online 05 May 2020

The original version of this Article contained an error in Figure 4, where the label of the X-axis ‘Size (nm)’ in panel (a) was incorrectly given as ‘Time (min)’. The original Figure 4 and accompanying legend appear below.

**Figure 4:**
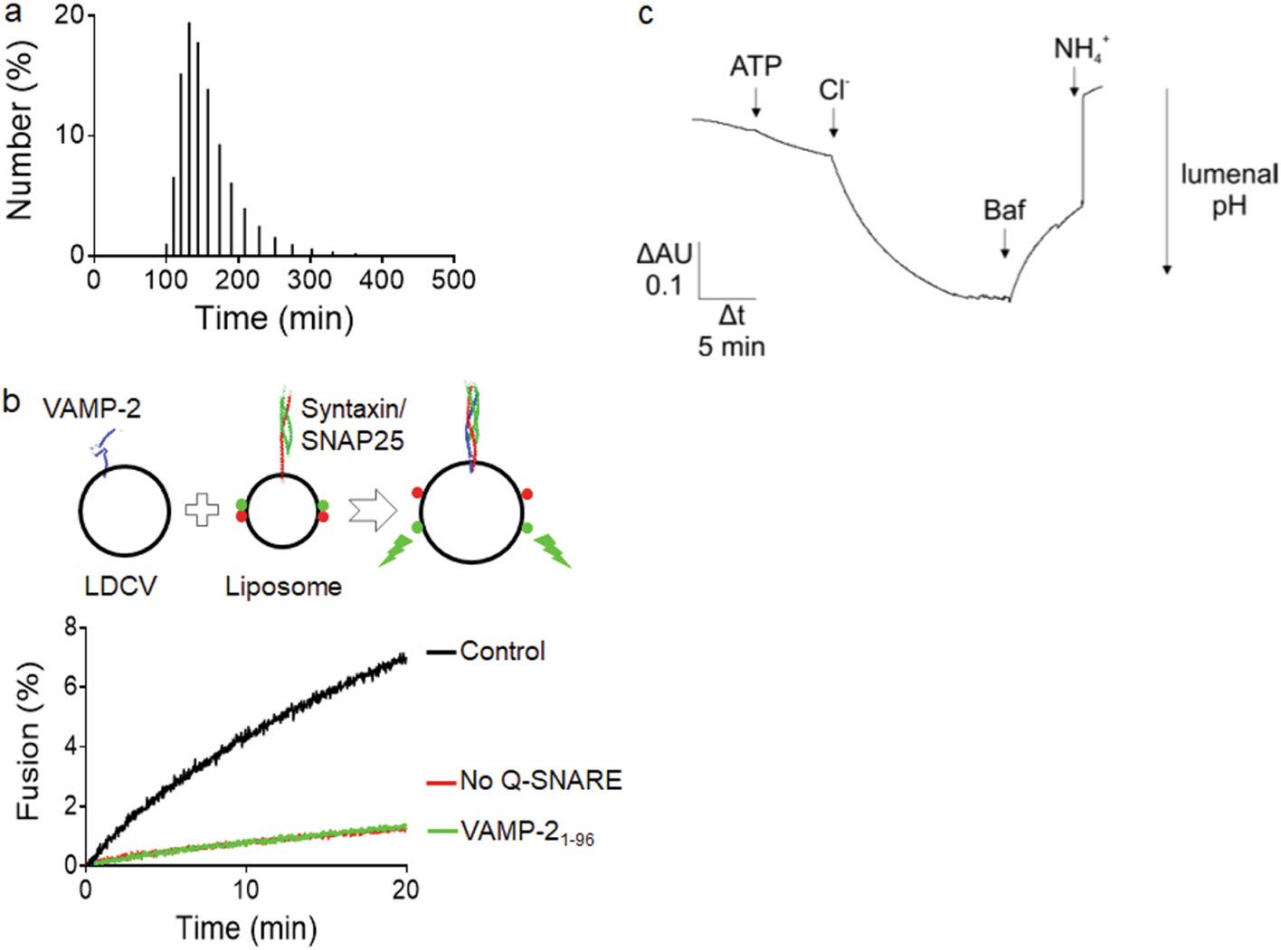
Biophysical and biochemical properties of purified LDCVs. (**a**) Size distribution of purified LDCVs analyzed using dynamic light scattering. The histogram shows the numbers as percentage of total. Average diameter of LDCVs is 153.7 nm, with a standard deviation (SD) of 42.2 nm. (**b**) Fusion of LDCVs with liposomes containing SNARE acceptor complexes. Fusion was measured by dequenching of labeled membrane lipids (lipid mixing)^18,22,28^ (top). Plasma membrane-mimicking liposomes contain phospholipids labelled with NBD (green fluorescence) and rhodamine (red fluorescence). The stabilized Q-SNARE complex (syntaxin-1A and SNAP-25A) called the deltaN complex^23^ is reconstituted in liposomes that mimic the plasma membrane. Fluorescence resonance energy transfer (FRET) between the two fluorophore-labeled lipids is reduced after LDCV fusion due to lipid dilution by unlabeled lipids of LDCVs, thus de-quenching the donor fluorescence. Soluble synaptobrevin-2 (VAMP-2_1-96_) and omission of SNAREs in liposomes prevented lipid mixing (bottom). Fluorescence values are normalized as a percentage value of the maximum donor fluorescence induced by 0.1% (vol/vol) Triton X-100 (TX-100) detergent treatment at the end of experiments. (**c**) ATP and Cl^-^ dependent acidification in LDCVs. LDCV acidification was monitored using 1 mM acridine orange as a reporter dye. The reaction was started by the addition of 1.2 mM MgATP followed by 50 mM KCl. Addition of 0.2 µM of the V-ATPase inhibitor Bafilomycin results in luminal re-alkalinization, indicating V-ATPase-specific proton pumping. The reaction was stopped by adding 15 mM (NH_4_)_2_SO_4_ which equilibrates the luminal pH with that of the medium.

The original Article has been corrected.

