# Supplementary material for: Isolation of large dense-core vesicles from bovine adrenal medulla for functional studies
Source: Sci Rep. 2020 May 5;10:7540. doi: 10.1038/s41598-020-64486-3 (PMC7200684; doi:10.1038/s41598-020-64486-3)

Isolation of large dense-core vesicles from bovine adrenal medulla for functional studies

Yelda Birinci[1], Julia Preobraschenski[2], Marcelo Ganzella[2], Reinhard Jahn[2,*], and Yongsoo Park[1,3*].

Full-length gels__Fig3a_SDHA

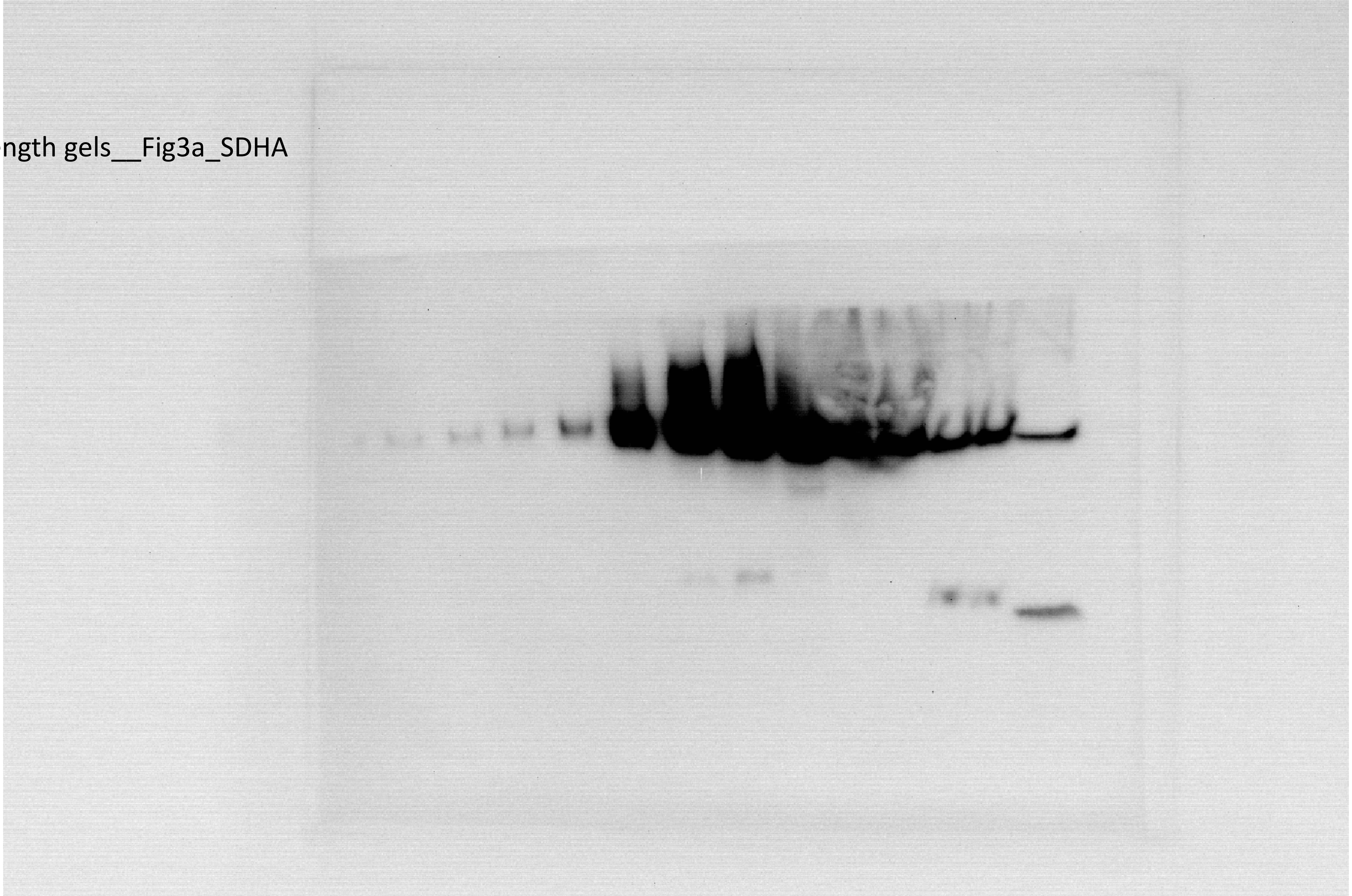

Full-length gels__Fig3a_VAMP2

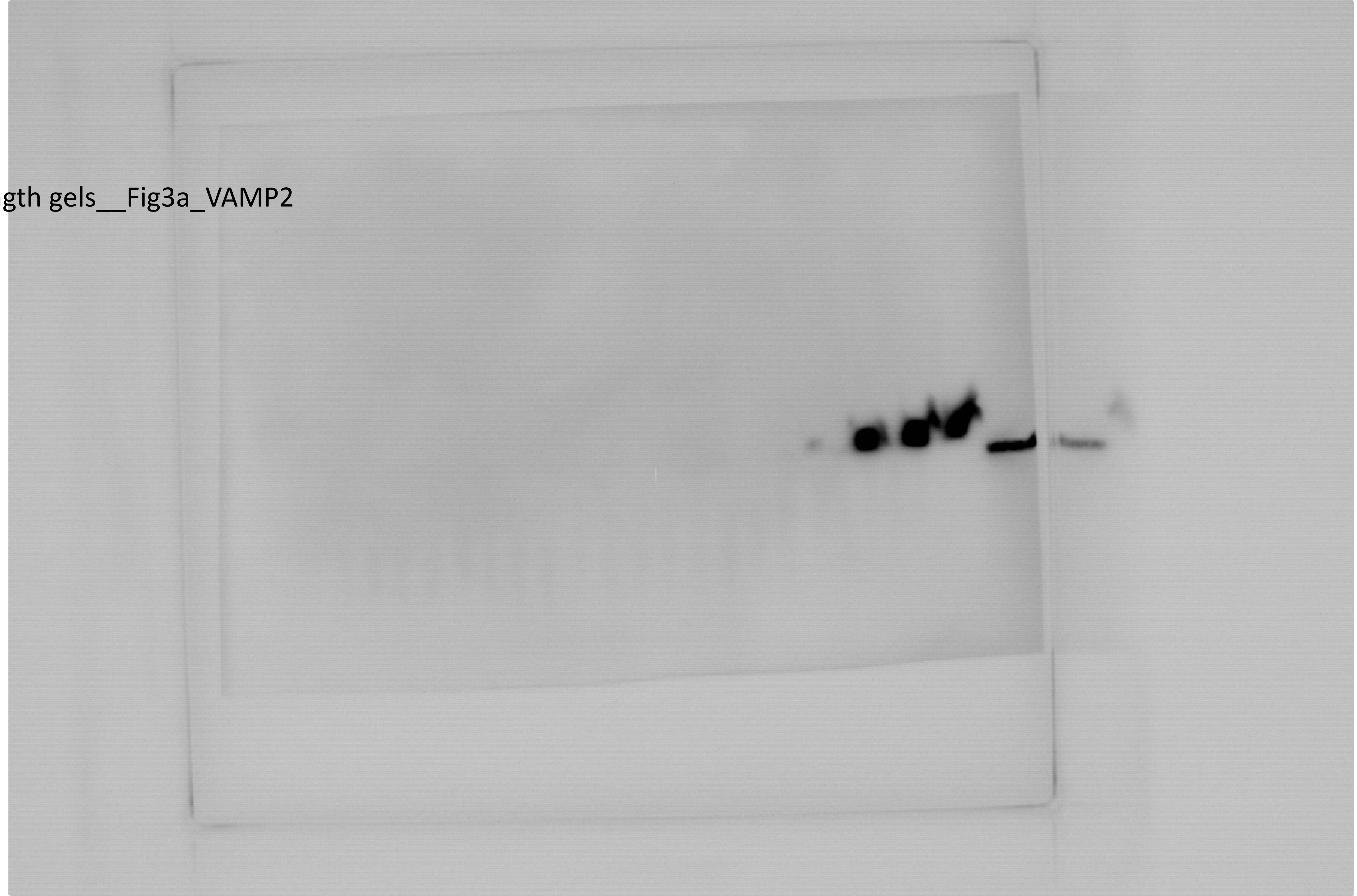

Full-length gels__Fig3b_SDHA

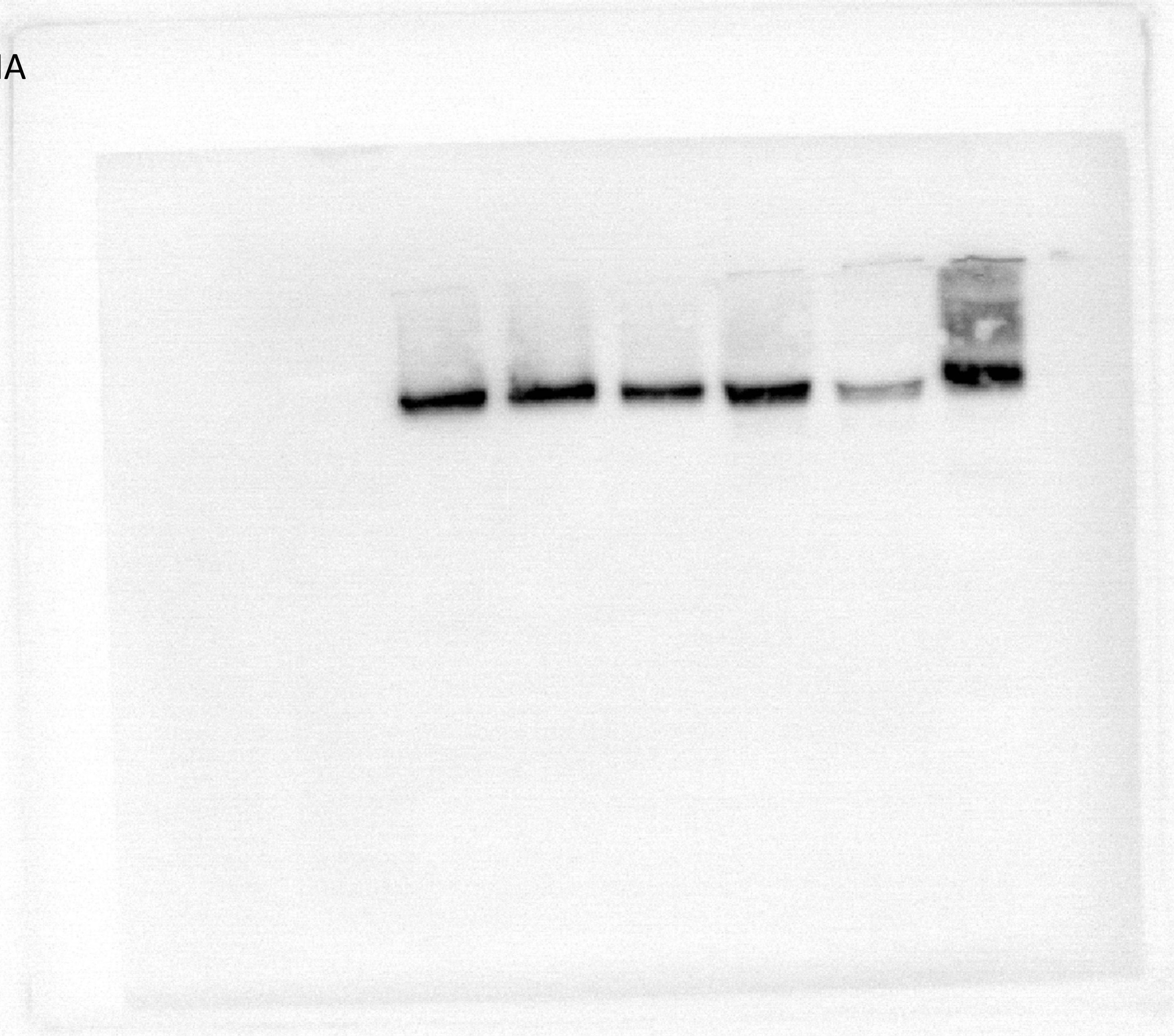

Full-length gels__Fig3b_VAMP2

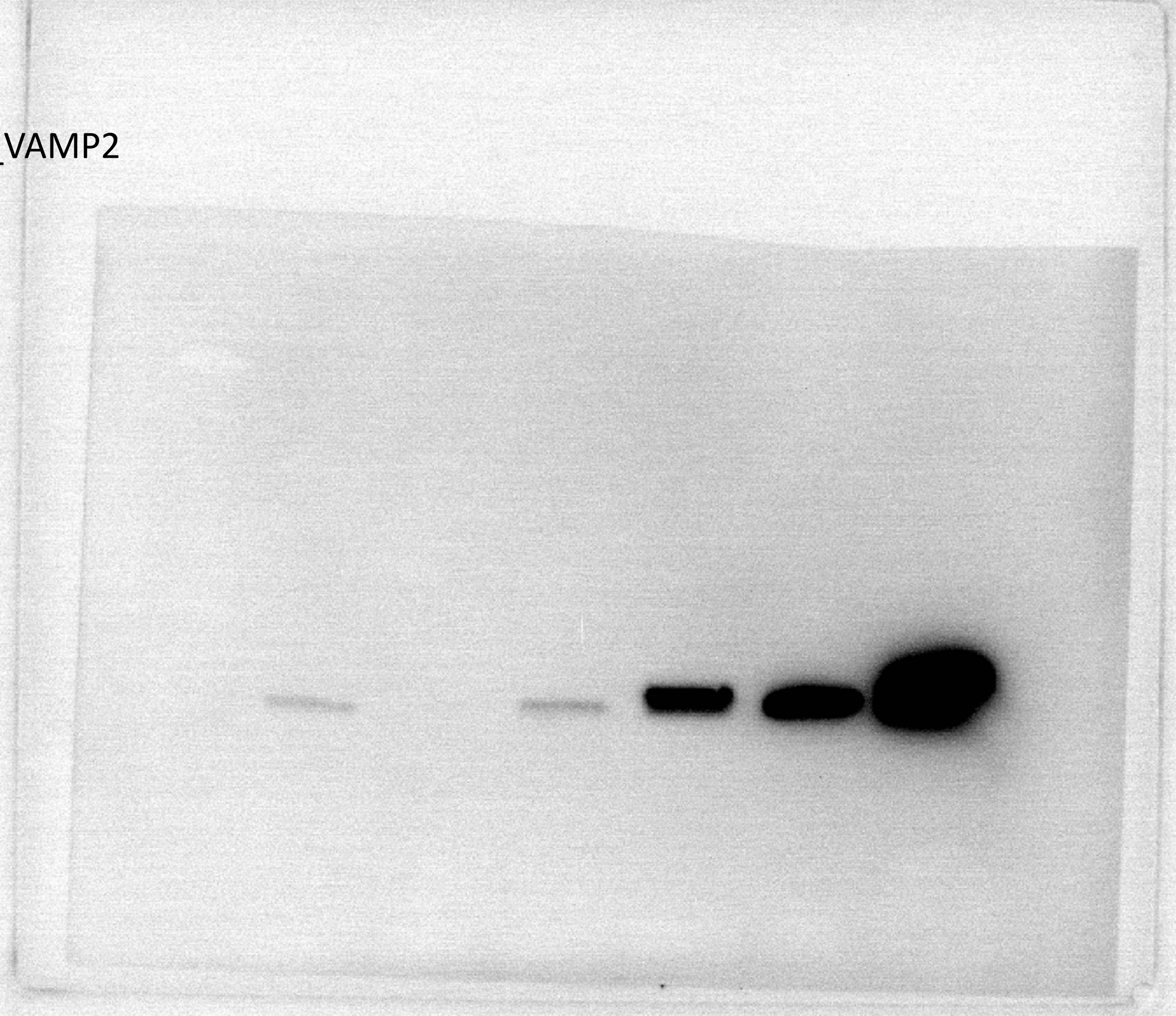

Full-length gels__Fig3c_DBH

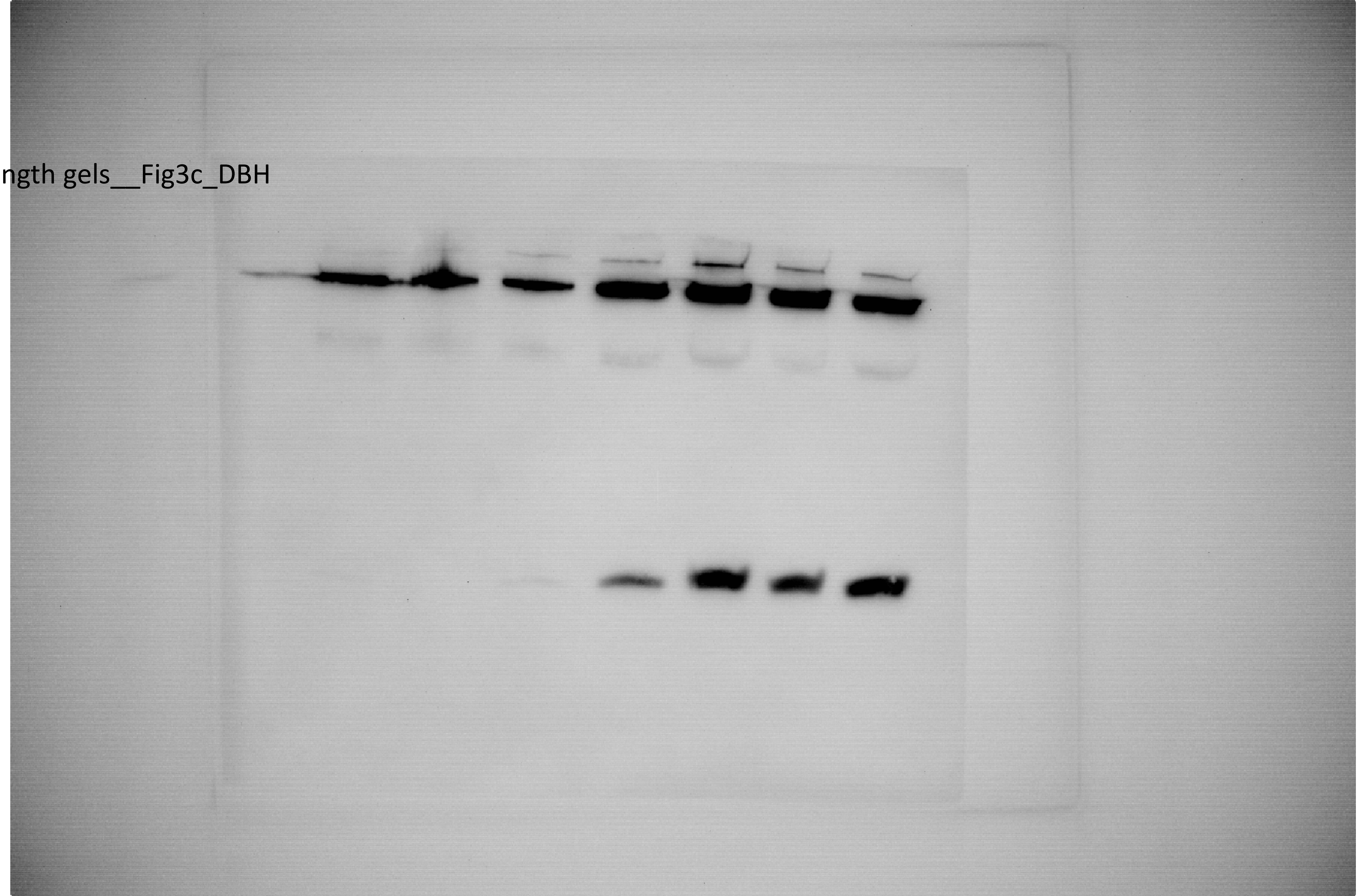

Full-length gels__Fig3c_SDHA

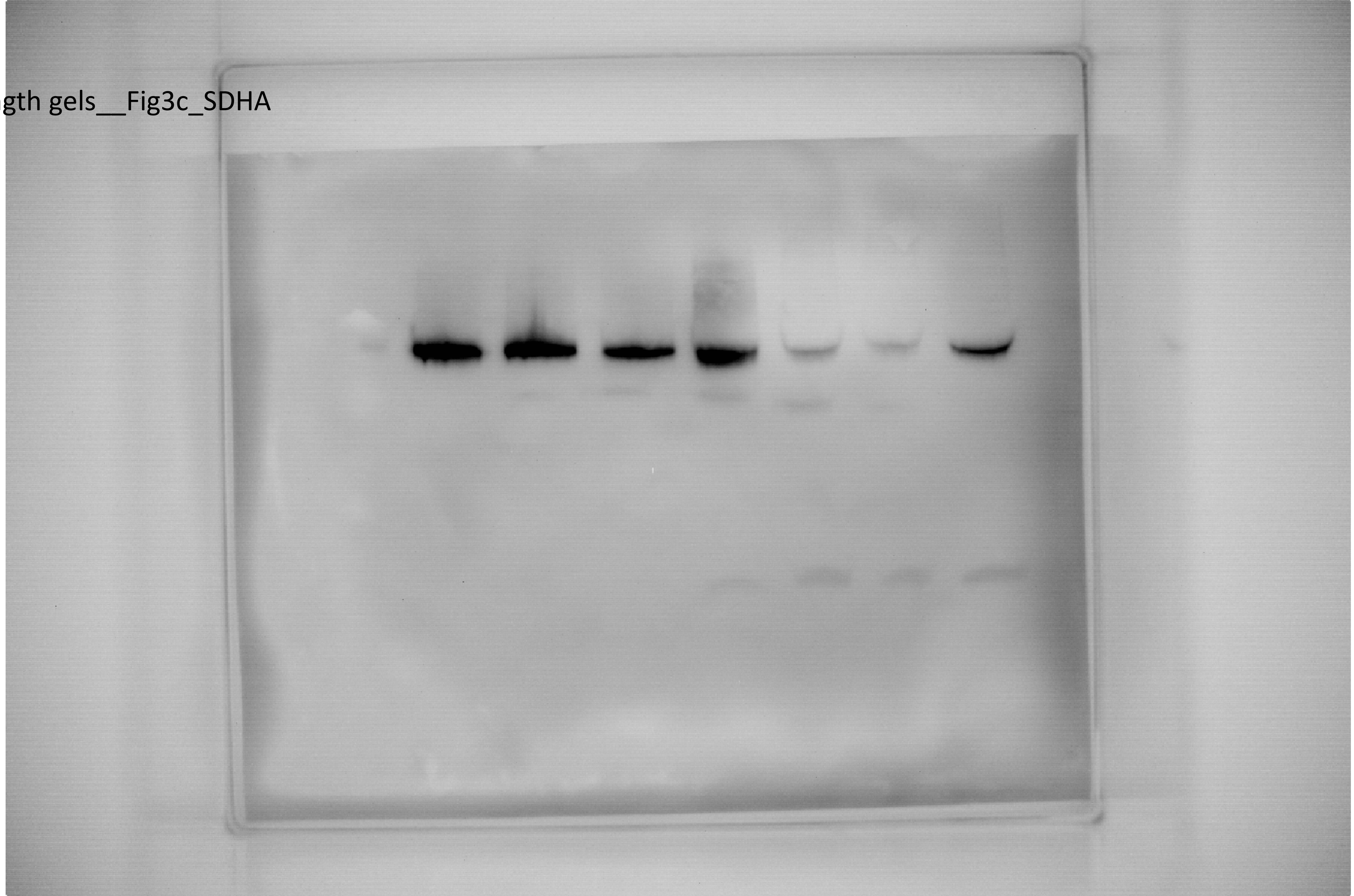

Full-length gels__Fig3c_VAMP2

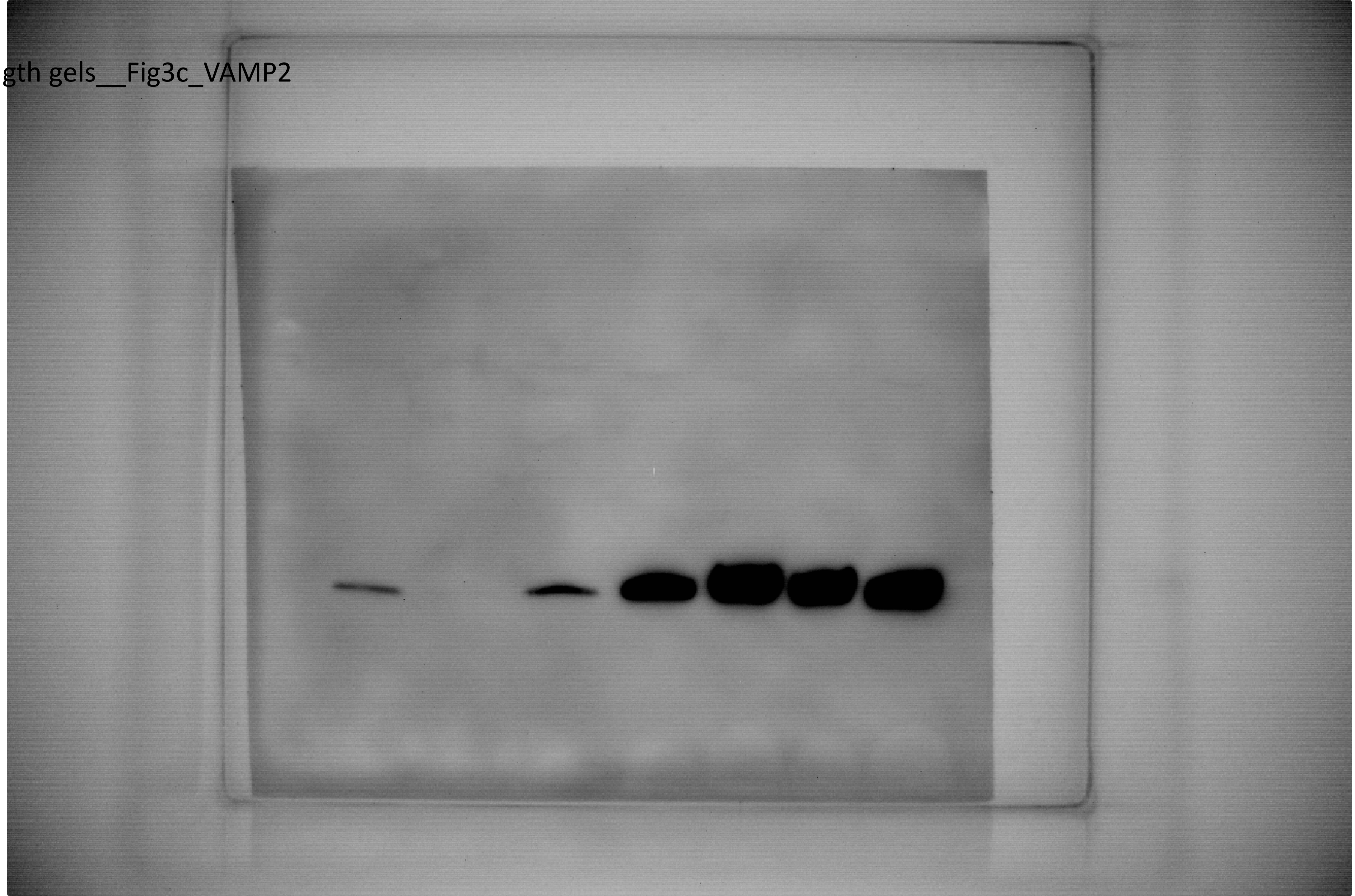

Supplement: Supplementary file 1 — Supplementary information. [file 41598_2020_64486_MOESM1_ESM.pdf]
